# Automated 3D light-sheet screening with high spatiotemporal resolution reveals mitotic phenotypes

**DOI:** 10.1242/jcs.245043

**Published:** 2020-06-01

**Authors:** Björn Eismann, Teresa G. Krieger, Jürgen Beneke, Ruben Bulkescher, Lukas Adam, Holger Erfle, Carl Herrmann, Roland Eils, Christian Conrad

**Affiliations:** 1 Division of Theoretical Bioinformatics, German Cancer Research Center (DKFZ), Heidelberg 69120, Germany; 2 Center for Quantitative Analysis of Molecular and Cellular Biosystems (BioQuant), University of Heidelberg, Heidelberg 69120, Germany; 3 Digital Health Center, Berlin Institute of Health (BIH)/Charité-Universitätsmedizin Berlin, Berlin 13353, Germany; 4 Advanced Biological Screening Facility Center for Quantitative Analysis of Molecular and Cellular Biosystems (BioQuant), University of Heidelberg, Heidelberg 69120, Germany; 5 Health Data Science Unit, Medical Faculty University Heidelberg and BioQuant, Heidelberg 69120, Germany; 6 Department for Bioinformatics and Functional Genomics, Institute for Pharmacy and Molecular Biotechnology (IPMB) Heidelberg University, Heidelberg 69120, Germany; 7 Heidelberg Center for Personalized Oncology, DKFZ-HIPO, DKFZ, Heidelberg 69120, Germany

**Keywords:** Cell cycle, High-content screening, Light-sheet microscopy

## Abstract

3D cell cultures enable the *in vitro* study of dynamic biological processes such as the cell cycle, but their use in high-throughput screens remains impractical with conventional fluorescent microscopy. Here, we present a screening workflow for the automated evaluation of mitotic phenotypes in 3D cell cultures by light-sheet microscopy. After sample preparation by a liquid handling robot, cell spheroids are imaged for 24 h *in toto* with a dual-view inverted selective plane illumination microscope (diSPIM) with a much improved signal-to-noise ratio, higher imaging speed, isotropic resolution and reduced light exposure compared to a spinning disc confocal microscope. A dedicated high-content image processing pipeline implements convolutional neural network-based phenotype classification. We illustrate the potential of our approach using siRNA knockdown and epigenetic modification of 28 mitotic target genes for assessing their phenotypic role in mitosis. By rendering light-sheet microscopy operational for high-throughput screening applications, this workflow enables target gene characterization or drug candidate evaluation in tissue-like 3D cell culture models.

## Abstract (precis)

**Highlighted Article:** A workflow using light-sheet microscopy to evaluate morphologic phenotypes in 3D cell cultures enables high-throughput studies of dynamic biological processes, target gene characterization and drug target candidate screens *in vitro*.

## INTRODUCTION

The cell cycle with its highly conserved and tightly regulated phases plays a key role in cancer development and progression. Cell cycle alterations are a hallmark of human tumors and many cell cycle proteins have oncogenic properties (Otto and Sicinski, 2017). Pharmacological or genetic modulation of mitotic oncogene expression is therefore a highly promising treatment approach.

To study dynamic biological processes such as the cell cycle *in vitro*, three-dimensional (3D) cell cultures provide a niche microenvironment that replicates the *in vivo* tissue more closely than traditional 2D methods (Pampaloni et al., 2007). Cellular and subcellular morphologies can thus be tracked in a physiologically relevant context, allowing characterization of therapeutic target gene function and evaluation of molecular perturbations. However, live fluorescent imaging of 3D tissue-like cell cultures with conventional laser scanning microscopes is problematic because of insufficient acquisition speed, low resolution in the Z direction, excessive light scattering within the tissue and high phototoxicity (Ntziachristos, 2010).

To overcome these challenges, recent advances in selective plane illumination microscopy (SPIM) or light-sheet microscopy provide imaging capabilities with increased acquisition speed, excellent optical sectioning and high signal-to-noise ratio (Kumar et al., 2014; Power and Huisken, 2017; Wu et al., 2013). Phototoxicity is reduced by separating excitation and detection axes, and exciting fluorophores in a single thin layer with a scanning Gaussian beam. SPIM thus enables the evaluation of phenotypes at the subcellular level in whole-spheroid or whole-organoid 3D cultures, with sufficient temporal resolution to visualize fast processes such as mitosis (Pampaloni et al., 2015; Strnad et al., 2016).

Although these features in principle make SPIM microscopes ideally suited to high-throughput or high-content screens, their distinct geometry and the large volumes of data generated pose new challenges for sample preparation as well as data processing and analysis (Preibisch et al., 2014; Schmied et al., 2016). Automated phenotype evaluation usually requires the delineation of imaged structures (segmentation) and their clustering into functional groups (classification) (Boutros et al., 2015). Classical machine learning methods such as random forests (RF) employ a user-defined set of features to categorize structured input data (Breiman, 2001; Ho, 1995). More recently, deep artificial neuronal networks such as convolutional neuronal networks (CNN) have emerged as promising alternatives (Krizhevsky et al., 2012). They can use unprocessed images as input and achieve image classification without the need for predefined features, often resulting in superior performance (Angermueller et al., 2016; Godinez et al., 2017; Pelt and Sethian, 2017; Van Valen et al., 2016); however, they require large annotated training data sets, which limits usability (Sadanandan et al., 2017).

Here, we describe a high-throughput screening workflow for the automated evaluation of mitotic phenotypes in 3D cultures imaged by light-sheet microscopy, from sample preparation to quantitative phenotype description. By using commercially available technology, this workflow is reproducible and easily adaptable to different cell culture models or molecular perturbations. A liquid-handling robot executes automated sample perturbation and mounting. Light-sheet imaging is performed with a dual-view inverted selective plane illumination microscope (diSPIM), a commercially available upright light-sheet system enabling high-throughput imaging of standard 3D cell cultures at isotropic resolution. A dedicated high-throughput image processing pipeline optimized for the diSPIM acquisition geometry combines convolutional neural network-based cell cycle phase detection with random forest-based classification to quantify phenotypic traits. Using this approach, we were able to detect mitotic phenotypes in 3D cell culture models following modulation of gene expression by siRNA knockdown or epigenetic modification. Our fully automated workflow thus adapts light-sheet microscopy for applications in high-throughput screening in 3D cell culture models.

## RESULTS

### Light-sheet imaging screen for high-content mitotic phenotype quantification

To evaluate the applicability of SPIM for high-throughput screening of mitotic phenotypes in 3D cell culture, we used an MCF10A breast epithelial cell line (Soule et al., 1990) stably expressing H2B-GFP to label DNA throughout the cell cycle. MCF10A cells provide an established and widely used model for benign breast tumors, with single MCF10A cells developing into multicellular 3D spheroids over the course of several days when seeded into laminin-rich hydrogel (Matrigel) (Debnath et al., 2003). We selected 28 mitotic target genes of interest for a high-throughput screen based on reported mitotic roles and a strong correlation (Pearson correlation >0.5) or anti-correlation (Pearson correlation <−0.5) of gene expression with altered methylation levels at one or multiple CpGs in the promoter or a distant regulatory genomic region, respectively (see Materials and Methods for details; Table S1). Target gene knockdown by siRNA transfection enabled us to analyze the effects of altered expression of these cancer-related genes in MCF10A cells. For the siRNA screen, two different siRNA were chosen per gene of interest and MCF10A H2B-GFP cells were transfected by solid-phase reverse transfection (Erfle et al., 2008). *INCENP* was used as a positive knockdown control because of its known severe effects on mitosis (Cai et al., 2018); non-coding siRNA was used as negative control.

**Fig. 1.**
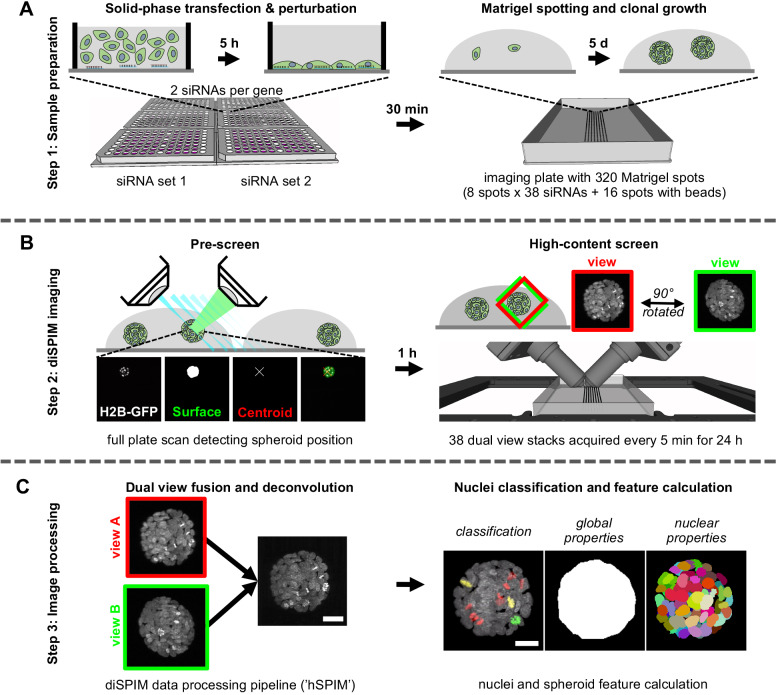
**Key steps of the light-sheet high-content live imaging screen.** (A) Step 1: Sample preparation. Cells were transfected in 2D by solid-phase reverse transfection in a 96-well plate format with two different siRNAs (siRNA set 1 and siRNA set 2) per target gene. Treated cells were then mixed with Matrigel and spotted in 0.2 µl droplets onto a one-well imaging plate. Over 5 days of culture, single cells clonally expanded into 3D spheroids. Each siRNA was analyzed in triplicate in three individual experiments. (B) Step 2: diSPIM imaging. In a low-resolution stage scan pre-screen, the positions of all spheroids were detected and samples selected for imaging by their position in the Matrigel spots; fused spheroids or cells growing on the plate in 2D were excluded. Subsequently, 38 individually treated samples were imaged every 5 min for 24 h by dual-view light-sheet microscopy, acquiring full stacks of view A (red) and view B (green) at a 90° angle to each other. (C) Step 3: Data processing. Raw image data were processed by fusing visual information of view A and view B. Processed data was further analyzed to evaluate the phenotype of each spheroid throughout the time lapse with regard to global spheroid features as well as single segment (single nucleus) properties. Scale bars:30 µm.

To achieve automated sample preparation, we developed a protocol for a liquid-handling robot, which mixes the pretreated cells with Matrigel and mounts them in small Matrigel spots in a defined grid on the imaging plate (Fig. 1A). This approach not only minimized the Matrigel volume to 0.2 µl per spot, reducing cost, but also ensured reliable positioning of samples with minimal pipetting variations or human errors.

After 5 days of cell culture at standard conditions, 3D spheroids were imaged with a diSPIM system for 24 h at 5 min time intervals (Fig. 1B). This choice of parameters accommodates the long cell cycle time (∼21 h) of MCF10A cells (Araujo et al., 2016) and enables single-cell tracking and the identification of subtle changes in the timing or morphology of nuclei undergoing mitosis.

Our imaging setup successfully realized the superior imaging capabilities of light-sheet microscopy over conventional confocal microscopes, including improved signal-to-noise ratio, higher imaging speed, better isotropic resolution and reduced light exposure compared with a spinning disc confocal microscope (Table S2). Reductions in image quality as a result of in-depth light scattering were negligible after dual-view image fusion (Fig. S1A). Due to the high temporal and spatial resolution, we were thus able to evaluate global, cellular and subcellular properties of mitotic gene knockdown phenotypes in live 3D spheroids (Fig. S1B-D).

We acquired live long-term image data for three spheroids per siRNA, amounting to 74 TB of raw data for a total of 228 spheroids. To address the challenge of data processing (Fig. 1C), we developed a high-throughput image processing tool called ‘hSPIM’, which was specifically tailored to the diSPIM geometry and acquisition properties. This pipeline allowed fast image fusion, deconvolution and data depth reduction of the raw diSPIM image data, based on a registration matrix and point spread function (PSF) detected from reference fluorescent beads. Sample background signal was reduced in our workflow by physically separating beads from spheroid samples; the registration matrix and PSF determined by imaging beads in Matrigel at a defined position was transferred to all acquired dual-view stacks for registration and deconvolution. Additionally, hSPIM enables basic single nuclei segmentation and textural feature detection. With most calculations executed in parallel on a high-performance GPU, we were able to process the raw data of one acquisition file within 8.23 s, reducing data size from 1.1 GB to 230 MB per position and time point (approximately 77 MB image data, 153 MB nuclei mask and 14 kB Haralick's texture features) while significantly improving XYZ isotropic resolution and signal-to-noise ratio (Fig. S1).

The processed image data was subsequently analyzed in a KNIME (Berthold et al., 2009) workflow. Key properties of individual spheroid development were quantified over time, including global descriptors of shape, volume and growth (e.g. total nuclei number and spheroid volume) as well as single nuclei-specific traits such as cell cycle phase and position within the spheroid (Table S3). To identify the cell cycle phase of each nucleus reliably, we compared the accuracy and performance of a random forest-based approach with a VGG-based convolutional neuronal network classifier (Simonyan and Zisserman, 2015) applied to the same manually annotated nuclei set (Fig. S2). The random forest classifier relied on Haralick's texture features (Haralick et al., 1973) calculated by the hSPIM processing pipeline and was able to detect the mitotic phase with 83% accuracy, whereas the CNN classified raw input image slices directly with 96% accuracy into the four key cell cycle stages (prophase, anaphase, metaphase, interphase) and was therefore chosen for all analyses.

### Detection of mitotic phenotypes in siRNA-treated MCF10A spheroids

**Fig. 2.**
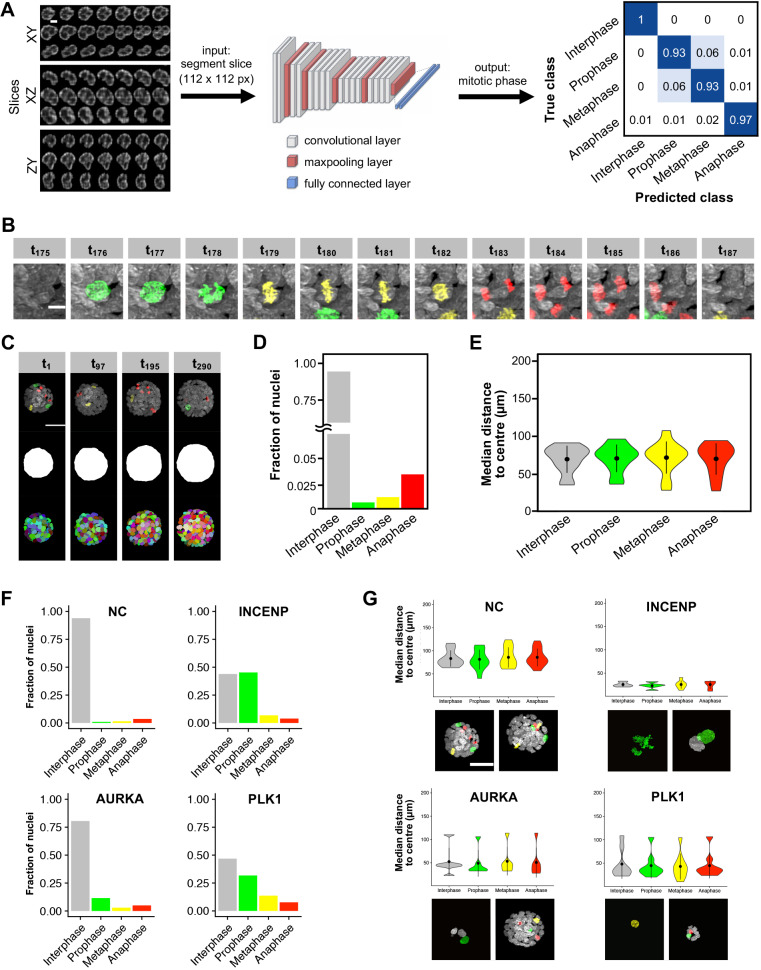
**Image analysis of diSPIM data to detect mitotic phenotypes in 3D spheroids.** (A) For cell cycle phase detection, 2D image slices of the 3D segments were used as input to a VGG-based convolutional neuronal network consisting of convolutional, maxpooling and fully connected layers as indicated (see also Fig. S2). The network outputs a probability for each of the cell cycle phases, with cross-correlation values shown as a measure of classification accuracy (cross-correlation values represent 10% of the manually annotated training data set, with the other 90% used for training the network). (B) Example time lapse (time points 175-187) of an untreated MCF10A cell undergoing mitosis, with interphase (white), prophase (green), metaphase (yellow) and anaphase (red) detected by deep learning image classification. (C) Four exemplary time points (t) of spheroid development imaged over 24 h (time points 1-290), with the classified cells (colors as in B), spheroid hull and segment maximum projection displayed. (D) Bar plot showing the total fraction of control nuclei detected in different cell cycle phases throughout the screen (*n*=205,068). (E) Violin plot depicting the distance of nuclei from the spheroid center during the different cell cycle phases (*n*=205,068). Black dots represent the median and whiskers the 25-75% interquantile range. (F) Examples of abnormal mitotic phase durations induced by different siRNAs. Bar plots show the total fraction of nuclei detected in different cell cycle phases for negative control samples transfected with non-coding siRNA (NC), as well as spheroids transfected with siRNAs against INCENP, AURKA and PLK1. (G) Examples of spheroid growth defects depicted by abnormal nuclei positions. Violin plots show the median distance of cells from spheroid centers during the different cell cycle phases for the same spheroids as in F. Black dots represent the median and whiskers the 25-75% interquantile range. Images show representative maximum and minimum sized spheroids at the start of the time lapse acquisition. Scale bars: 5 µm (A,B), 50 µm (C,G).

Accurate cell cycle phase detection by CNN classification (Fig. 2A) and the high temporal resolution of the diSPIM screen allowed us to track single cells through the different phases of the cell cycle (Fig. 2B,C). In non-transfected control samples, 94.7% of all nuclei throughout the time lapse were classified as interphase, 0.9% as prophase, 1.2% as metaphase and 3.2% as anaphase (Fig. 2D). As the high isotropic spatial resolution allowed us to determine nuclei and spheroid volumes at different stages, we also confirmed that prophase nuclei were on average the largest, followed by inter- and metaphase nuclei. Although it has been suggested that nucleus position within the tissue influences cell cycle fate (Pettet et al., 2001), we registered a similar radial distribution of cells in all cell cycle phases within MCF10A spheroids at day 6 of clonal development (Fig. 2E).

In siRNA-transfected samples, we observed a wide range of mitotic phenotype alterations. An over-representation of different cell cycle phases compared with control MCF10A spheroids indicated cell cycle arrest; *INCENP* and *AURKA* knockdown spheroids, among others, showed more cells in prophase (Fig. 2F), whereas *MYC* and *ATOH8* knockdown resulted in more anaphase cells. *PLK1* knockdown nuclei displayed an increase in all mitotic classes, suggesting an elongated cell cycle (Fig. 2F). Apoptotic cells frequently led to the assignment of improper mitotic phase transitions (such as prophase to interphase) in *PLK1*, *EME1* and *CEP85* knockdown spheroids. Defects in spheroid growth were identified in *INCENP*, *AURKA* and *PLK1* knockdown spheroids, among others, whereas we did not observe abnormal positioning of cells in individual cell cycle phases (Fig. 2G).

**Fig. 3.**
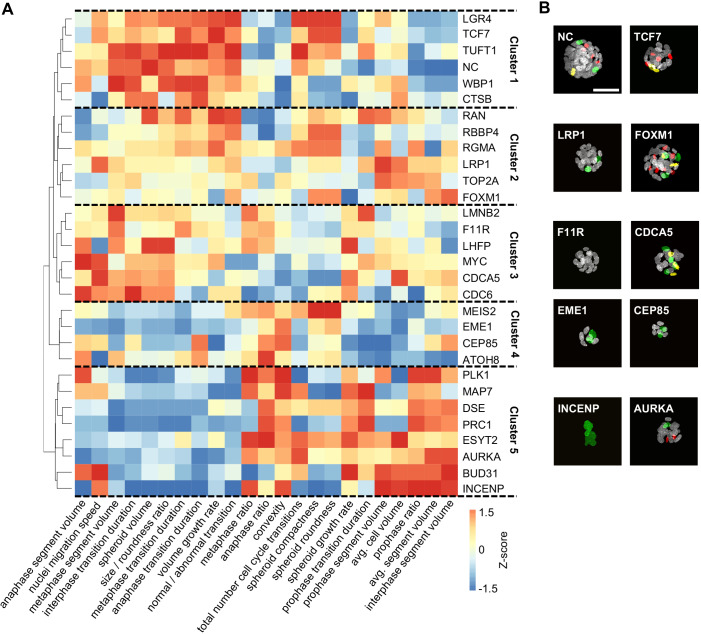
**Clustered phenotype analysis of all features detected in diSPIM high-content screen.** (A) Rank-based hierarchical clustering of siRNA knockdown mitotic phenotypes by features describing the global and nuclei-specific properties results in distinct clusters of siRNA target genes, with the number of clusters chosen based on qualitative assessment of morphologic similarity within groups. (B) Example images of spheroids from each cluster with classified nuclear mitotic phases (white, interphase; green, prophase; yellow, metaphase; red, anaphase). Scale bar: 50 µm.

Hierarchical clustering of all mitotic phenotype quantifications (Table S3) distinguished five major phenotypic groups (Fig. 3). Cluster 1 comprised spheroids with a high growth rate closely resembling that of the noncoding siRNA control. Cluster 2 mostly contained samples with increased nuclear volumes during prophase and a higher proportion of cells in this phase, indicating prophase arrest and formation of macronuclei with increased DNA content. A higher volume growth rate was detected in spheroids in cluster 3, with some knockdown target genes (*LMNB2*, *F11R* and *LHFP*) also resulting in larger nuclei during anaphase. Cluster 4 spheroids showed strong phenotypes with reduced spheroid volume growth and a low total number of cell cycle transitions, indicating diminished mitotic activity. Finally, cluster 5 spheroids showed aberrant phenotypes in several features, describing grave cellular and mitotic defects.

To evaluate the performance of the diSPIM screening workflow and analysis pipeline, we compared detected phenotypes to the MitoCheck database assembled from imaging the first two to four cell divisions of HeLa H2B-GFP cells upon siRNA target gene knockdown (Cai et al., 2018). Notably, despite the use of different cell lines, all of the strong phenotypes identified in our study were also described in the MitoCheck screen, including reduced spheroid growth in *EME1* and *ATOH8* knockdown cultures, elongated cell cycle phases in *ESYT2* and *PLK1* knockdown cells, as well as cell cycle arrest and increased apoptosis in *INCENP*, *MAP7*, *DSE* and *PRC1* knockdown cells. Minor phenotypes such as macronuclei formation in some spheroids with *CEP85* or *MEIS2* knockdown and interphase arrest induced by *MYC* siRNA transfection were not detected by the MitoCheck screen.

### Detection of mitotic phenotypes after epigenetic perturbation

To demonstrate the utility of our workflow, we used a novel molecular tool based on the CRISPR/Cas system (Cong et al., 2013; Jinek et al., 2012) to target regulatory epigenetic elements of the selected mitotic genes. Fusion proteins of deactivated Cas9 with no endonuclease activity (dCas9) with the effector domain of methylome-modifying proteins (dCas9-ED) have been shown to alter the epigenome at a targeted genomic location defined by an appropriate single guide RNA (sgRNA) (Gilbert et al., 2013; Pulecio et al., 2017). We designed fusion proteins of dCas9 and the effector domain of either DNMT3a methyltransferase to achieve CpG methylation or TET1 for demethylation (Fig. S3). A dCas9 without added effector domain was used as a control, physically blocking binding sites for regulatory factors. As MCF10A cells are highly resistant to plasmid transfection, we used human embryonic kidney 293 (HEK293) cells, which also develop into multicellular spheroids when seeded in Matrigel, and generated cell lines stably expressing the different constructs.

In a 2D pre-screen, we detected overall low effectivity of the dCas9-ED fusion proteins across our set of target genes (see Materials and Methods for details; Fig. S4), but identified the target gene *RGMA* as robustly showing a mitotic phenotype under different methylome modifying conditions (dCas9-DNMT3a with sgRNAs targeting anti-correlated CpGs or the transcription start site (TSS), and dCas9-TET1 with sgRNAs targeting correlated CpGs). We therefore selected *RGMA* for 3D screening of mitotic phenotypes upon epigenetic modification, using the same sample preparation and imaging workflow as described above for the siRNA screen. At 3 and 5 days after sgRNA plasmid transfection, every sgRNA-transfected spheroid (as assessed by GFP expression) was imaged and evaluated by Hoechst staining for mitotic phenotypes.

**Fig. 4.**
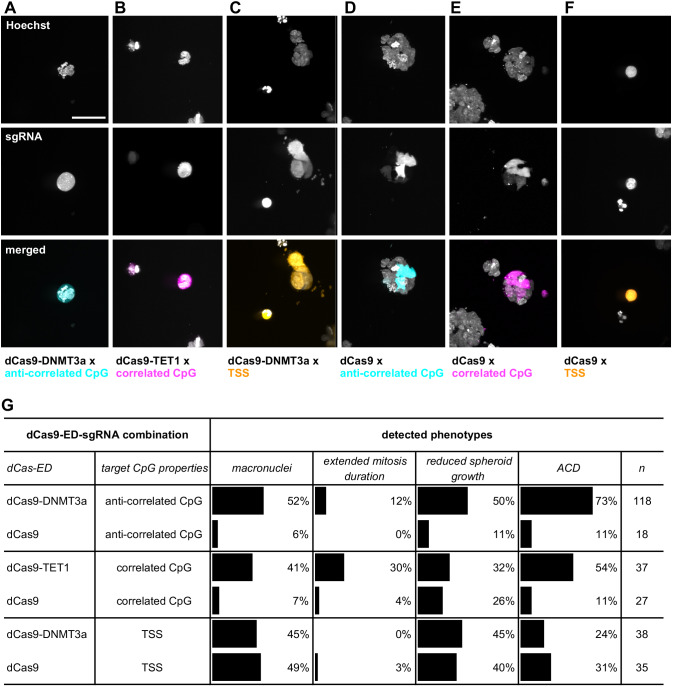
**Phenotype analysis of dCas9-ED targeting RGMA regulatory CpGs in 3D HEK293 spheroids.** Example images of HEK293 spheroids stably expressing dCas9-ED or dCas9, after transfection with sgRNA designed to epigenetically alter RGMA expression, in the following combinations: (A) dCas9-DNMT3a with sgRNA targeting anti-correlated CpG (cyan), (B) dCas9-TET1 with sgRNA targeting correlated CpG (magenta), (C) dCas9-DNMT3a with sgRNA targeting the TSS (orange), (D) dCas9 with sgRNA targeting anti-correlated CpG (cyan), (E) dCas9 with sgRNA targeting correlated CpG (magenta) and (F) dCas9 with sgRNA targeting the TSS (orange). (G) Summary of phenotypic effects of epigenetic targeting of RGMA expression in HEK293 spheroids, showing the percentage of spheroids displaying abnormal cellular and global properties, including macronuclei formation, extended mitosis duration, reduced spheroid growth and apoptotic condensed DNA (ACD). Scale bar: 50 µm.

Most prominently, and in agreement with the MitoCheck database, macronuclei were detected in 41-52% of all spheroids transfected with either dCas9-DNMT3a and sgRNA targeting anti-correlated CpGs (Fig. 4A), dCas9-TET1 and sgRNA targeting correlated CpGs (Fig. 4B), or dCas9 and dCas9-DNMT3a with sgRNA locating to the TSS (Fig. 4C,F). Transfection with dCas9 control protein and sgRNA targeted to regulatory CpGs resulted in macronuclei in only 7% (anti-correlated CpGs) or 6% (anti-correlated CpGs) of spheroids (Fig. 4D,E), confirming the specificity of our epigenetic modification of *RGMA*. Reduced spheroid growth, apoptotic condensed DNA and elongated mitosis or mitotic arrest were also frequently observed, indicating that *RGMA* knockdown has severe effects on cellular homeostasis (Fig. 4G).

Modulation of *RGMA* gene expression at the transcriptional level, using a dCas9-ED system, and at the translational level, using siRNA, thus results in similar mitotic phenotypes, which can be detected with our high-throughput light-sheet imaging workflow, illustrating its versatility.

## DISCUSSION

The high-throughput light-sheet live imaging workflow presented here provides a novel tool for screening individually treated 3D cell cultures with high spatial and temporal resolution, signal-to-noise ratio, fast acquisition speed and minimal phototoxic effects. Automation of the different steps from sample treatment and mounting to spheroid position detection and image acquisition, as well as the commercial availability of all materials, ensure that the workflow is reproducible and applicable to different culture models or treatment methods. Furthermore, we provide an easy-to-use image processing pipeline adapted to the geometry of the dual-view inverted light-sheet system, using a CNN for reliable cell cycle phase classification in 3D, which we have made available to the community online.

By applying this workflow to 3D cultured MCF10A H2B-GFP cells transfected with siRNA targeting mitotic genes, and HEK293 cells transfected with dCas9-based epigenetic modifiers, we were able to evaluate the effect of single gene knockdown on key cellular and spheroid features. Our integrated high-content analyses also highlight similar phenotypes caused by different genes. Due to the superior temporal and spatial resolution provided by the diSPIM system in combination with long-term acquisition, we could track cells over 24 h and detect subtle mitotic 3D phenotypes not accessible with conventional fluorescent microscopy.

Because the diSPIM geometry uses dipping lenses, phenotypes that can be evaluated by high-throughput live imaging with this workflow are restricted to intracellular perturbations such as siRNA- or CRISPR-Cas9-based screens, although an end-point analysis of fixed samples can also be conducted. To extend this workflow to other applications, such as using small molecule libraries for high-throughput drug screens, samples need to be physically separated into distinct wells during culture. Novel cell culture plate formats could accommodate this, for example by mounting spheroids on invertible pillar structures for culture in multiwell plates.

The light-sheet imaging setup presented here is thus adaptable for high-throughput screening of 3D cell cultures in a variety of settings. As spheroids can be recovered after imaging, it is also compatible with combined approaches correlating image data with other modalities, including single-cell genomic or transcriptomic analyses. We therefore expect that the ability to evaluate 3D phenotypes quantitatively in live cell cultures at high throughput will advance functional characterization of dynamic cellular processes in tissue-like microenvironments, in cancer research and beyond.

## MATERIALS AND METHODS

Please find methods and material-related hardware, software and wet lab items additionally listed in Table S4.

### Culture of MCF10A H2B-GFP cells

MCF10A cells (CRL-10317, ATCC, Manassas, VA) transfected with a pBabe vector containing a construct of GFP-labelled H2B were kindly provided by Zev Gartner and colleagues (University of California, San Francisco). MCF10A H2B-GFP cells were between passage numbers 25 and 31 and routinely tested for contamination. Cells were cultured in 2D in 25 cm^2^ culture flasks (Greiner bio-one, Kremsmünster, Austria) in DMEM/F12 medium (ThermoFisher Scientific #11039, Waltham, MA) supplemented with 5% horse serum, 10 µg/ml insulin (Life Technologies, Carlsbad, CA), 20 ng/ml EGF, 0.5 mg/ml hydrocortisone and 100 ng/ml cholera toxin (Sigma-Aldrich, St Louis, MO) under standard culture conditions (5% CO_2_, 37°C), and passaged after reaching 80-90% confluency with 0.05% trypsin (Life Technologies) every 3-4 days.

### Solid-phase reverse siRNA transfection

Solid-phase reverse transfection siRNA transfection mix was prepared as described (Erfle et al., 2008), but using trehalose dihydrate (Merck #T9531, Darmstadt, Germany) instead of sucrose. For transfection, trypsinated MCF10A H2B-GFP cells were diluted in growth medium to a density of 5×10^5^ cells/ml. Cell suspension (10,000 cells in 100 µl) wase added to each well of the solid-phase reverse transfection mix. After 5 h, cell medium was removed and the cells resuspended by directly adding 50 µl of 0.25% trypsin (Life Technologies #25200056) to each well.

### High-content cell spotting in Matrigel

Mixing of cells with Matrigel (Corning, New York, NY) and spotting into OneWell plates (Greiner bio-one CELLSTAR OneWell Plate #670180) was conducted by an automated liquid-handling robot from Hamilton Robotics with a custom protocol. In short, from each cell suspension transfected with individual siRNA, 60 isolated cells in 3 µl medium were mixed with 10 µl Matrigel. Subsequently, each mixture was spotted eight times with a single spot volume of 0.2 µl in an array of two columns by four rows, resulting in a total of 320 spots in 40 columns and 8 rows on the imaging plate. One subarray of spots was always dedicated to beads (ThermoFisher Scientific #7220) mixed with Matrigel, used for registration of the two acquired views. Positioning of each spot was identical with the positions of a standard 1536-well plate. After 10 min at 37°C for Matrigel solidification, culture medium was added and the samples incubated under standard culture conditions until imaging.

### diSPIM imaging

Imaging was conducted with a dual-view inverted selective plane illumination microscope (diSPIM) as described (Kumar et al., 2014). The microscope was equipped with LMM5 laser (Laser Illumination Laser Merge Module 5, Spectral Applied Research, Richmond Hill, Canada) and Quad Filterset (F59-405/F73-410/F57-406) purchased from AHF (Tübingen, Germany). Images were acquired by two water-cooled ORCA-Flash4.0 Hamamatsu sCMOS cameras (Hamamatsu, Japan). Cooling was provided with Julabo F250 cooling circuit (Julabo, Seelbach, Germany). Standard culture conditions were provided by an incubation chamber (3i ECS2) from Intelligent Imaging Innovations (Denver, CO); direct airflow over the SPIM head was minimized to avoid unnecessary vibrations. All imaging time lapse acquisitions were conducted with 320 µW laser power for 488 nm excitation wavelength (measured at the sample). Readjusting the fine alignment of the microscope was conducted shortly before start of the acquisition.

### Low resolution pre-screen

To detect each spheroid's positions and select the spheroids to be imaged, we conducted a fast, low resolution stage-scan pre-screen. A grid of imaging positions was defined across the imaging plate, with each position placed at the center of one column of spots. As the automated spotting process resulted in spots with defined positions and sizes, we were able to use the same grid of stage scan acquisition positions repeatedly for every pre-screen. Potentially caused by small manufacturing differences in the imaging plate, we solely needed to adjust the general Z-position off-set, underlining the robustness of our sample preparation process. Each position acquisition resulted in a X_microscope_-Stack of 1,200 slices with a step size of 5 µm, a pixel resolution of 0.648 µm/px and a field of view of 333 µm.

Acquisition of the pre-screen took 31 min and produced 96,000 images. This pre-screen data was subsequently analyzed by a KNIME image processing workflow detecting the XYZ_microscope_ position, size and shape of each cell cluster. Per spot, we detected an average of 2.6 spheroids. Small, flat and elongated cell clusters were excluded and the remaining spheroids ranked based on their Z-position. To minimize obstructions in the illumination and detection path and maximize image quality, the spheroids with the largest Z-coordinates were selected for imaging for each condition.

Defined cell spheroids (*n*=38) plus two positions with fluorescent beads (used for image processing) were imaged with the diSPIM system for 24 h at maximal temporal and spatial resolution for treatment evaluation.

### Position scan acquisition

Preselected positions from the KNIME analysis of the pre-screen were checked and, if necessary, manually corrected. An additional registration position was added as first and last position, using imaging beads mixed in Matrigel.

Imaging parameters for dual-view synchronous piezo/slice scan (stack acquisition) were set to acquire two stacks of 1024 px^2^ in XY_image_ with the maximal camera resolution of 0.1625 µm/px centered to the field of view of the camera and 260 slices in Z_image_ with a slice interval of 0.5 µm starting with view A (right camera acquisition). Sample exposure was set to 1.5 ms and the option for ‘Minimized slice period’ enabled. The option for ‘Autofocus during acquisition’ was enabled with the autofocus running on the registration position imaging beads every acquisition cycle, with 40 slices acquired every 0.5 µm. The off-set was detected by the ‘Vollath’ algorithm.

Because of the acquisition limitations of the microscope of a minimal 4-5 s per position scan and stage repositioning, we imaged at intervals of 5 min for 24 h, resulting in a total data volume of 10.06 TB, which was stored locally. The high temporal resolution was essential for tracking nuclei as they progressed through the cell cycle. Throughout time lapse acquisition, we did not need to adjust for any position off-set introduced by deformation of the Matrigel or external influences as samples remained almost universally in the field of view.

### Image processing

Raw data was processed by a custom software, hSPIM, specifically adapted to the geometry of the diSPIM and the separately acquired registration beads positions. In hSPIM, the registration matrix of the two views is detected for each time point of the screen by registration of beads in 3D. Additionally, the PSF is extracted. This registration matrix and PSF are stored and used for registration and deconvolution of all other acquired positions at this time point. Furthermore, the software performs a segmentation of the nuclei, from which different geometrical and textural features are extracted for each segment. Deconvolved fusion images and segment images as well as segment and feature table are stored and used for further image analysis. In addition, the hSPIM software can directly visualize a registered and deconvolved image snapshot in 3D, store a view angle, and export a 3D movie of a single position. Library code and documentation for hSPIM are available at https://github.com/eilslabs/diSPIM_screen.

### High-content KNIME analysis workflow

Following the raw image processing, we developed a KNIME workflow to analyze key cellular and global properties of each spheroid throughout the acquired time lapse. The workflow is available at https://github.com/eilslabs/diSPIM_screen.

#### XYZ_microscope_ displacement

By tracking the positions of single beads over time, we could detect and correct for the global offset in all dimensions of the microscope introduced through fine displacements of the imaging plate or expansion of the diSPIM components.

#### Clustering of segments into spheroids

To segregate segments from two spheroids acquired at a single imaging position into individual spheroid clusters, we analyzed the geometric distance of each segment to all others and clustered segments accordingly.

#### Spheroid size

The clustering enabled us to combine all segments of one spheroid and determine spheroid size.

#### Cell cycle phase classification

For precise cell cycle phase classification, we used a VGG-based convolutional neuronal network trained on a set of manually classified images. The CNN calculated the probability for each of the four cell cycle phases (interphase, prophase, metaphase, anaphase) for each XY, XZ and YZ slice. The class with the highest sum in likelihood for each segment was selected as the cell cycle phase of the nucleus at this time point.

#### Geometric nuclei class features

Single nucleus size, intensity, position of segments from the center of the spheroid and predicted cell cycle class were recorded over time.

#### Nuclei migration speed

By tracking the position of each segment over time, we analyzed the median migration speed of all cells in each spheroid.

#### Time lapse movie

For individual evaluation, we exported the maximum projected time lapse movie of each position, including cell cycle classification and spheroid hull.

### Spheroid feature evaluation

Selected image features quantified by the KNIME workflow (Table S3) were subjected to further quantitative analysis in R. Additional quantitative features such as mean cell volume (estimated as the ratio of spheroid volume to nuclei number) and average nucleus size in different cell cycle phases were computed. The fraction of cells detected in different cell cycle phases was averaged across all time points. To calculate instantaneous spheroid growth rates from nuclei numbers, the number of nuclei over time was smoothed using the lowess function with parameters *f*=1/3, iter=3L, delta=0.01×diff(range(NrCells[1:n.rows[[pos]],pos])), and differentiated using the diff function.

All feature measurements from all plates were combined into one matrix, centered by subtracting the column means from their corresponding columns, and scaled by dividing the centered columns by their standard deviations. As mechanical plate drift resulted in spheroids lying partially outside the field of view in one plate, affected features (nuclei and spheroid growth rate, compactness, convexity, sphericity, spheroid volume and cell volume) were excluded for this plate.

To identify clusters of siRNAs causing similar phenotypes, rank-based clustering was performed using the rank, dist, and hclust functions. The number of clusters was chosen based on qualitative assessment of morphologic similarity within groups. Heatmaps were created using the heatmap.2 function from the gplots package or the aheatmap function from the NMF package.

### Statistical analysis

Because images were acquired of samples cultured in one-well plates with homogeneous culture conditions, no randomization of siRNAs across the culture plate was necessary. Investigators were not blinded during experiments or analysis. No statistical tests were used during data analysis.

### dCas9-effector domains construct synthesis

We fused the catalytic C-terminal effector domains of epigenome modifying enzyme (DNMT3a, TET1) C-terminally to the dCas9 via a linker and added two nuclear localization sequences (NLS) for improved nuclear localization and M2-Flag to the N terminus. A dCas9 with no C-terminal addition of an ED was used as binding control and to block binding sites physically for regulatory factors. Source constructs were obtained from AddGene (Watertown, MA) for dCas9 (Gilbert et al., 2013), DNMT3a (Vojta et al., 2016) and TET1 (Tahiliani et al., 2009). dCas-ED constructs (C49–dCas9; C54–DNMTA3-dCas9; C57–TET1-dCas9) were assembled by Gibson Cloning (NEB #E2611) following manufacturer guidelines. Linker (GGGGS), NLS (PKKKRKV) and M2-Flag (DYKDHDG) DNA sequences, as well as adapter primers were ordered from Eurofins Genomics. Successful cloning was assessed by sequencing by GATC Biotech AG, western blot (M2-Flag) and expression in HEK293 cells detected by immunostaining. Plasmid maps and construct components are available from the authors on request.

### Stable dCas9-ED expression in HEK293 cell line

HEK293 cells (5×10^5^) were transfected with 5 µg plasmid DNA of the different dCas9-ED constructs (C49, C54, C57) with Lipofectamine 2000 (Invitrogen, Carlsbad, CA) following manufacturer guidelines. At 48 h post dCas9-ED plasmid transfection, transfected cells were selected by addition of G418 (Geneticin, Sigma-Aldrich) antibiotic to the culture medium at a concentration of 500 µg/ml. Stable dCas9-ED expressing cell lines were frozen after four passages under G418 selection.

### CpG selection

We targeted the different epigenome modifying molecular tools to specific genomic sites by combining different sgRNAs with the different effector domains to modify distal or proximal regulatory CpGs with regulatory properties (Table S1). Based on a data set comprising 450k Illumina gene expression and CpG methylation data from human breast cancer patients (Parashar et al., 2018), available on the UCSC genome browser (Kent et al., 2002), we selected CpGs with high correlation (Pearson correlation >0.5) or high anti-correlation correlation (Pearson correlation <−0.5) between CpG methylation level and target gene expression. We detected up to seven correlated or anti-correlated regulatory CpGs per target gene. Correlated CpGs (low CpG-me results in reduced expression) are expected to result in a gene knockdown phenotype when targeted by the TET1 dCas9-ED, whereas anti-correlated CpGs (high CpG-me results in reduced expression) are expected to show the abnormal mitotic phenotype when targeted by DNMT3A. Target genes that had only a single regulatory CpG with a correlation between expression level and CpG methylation below 0.6 were not further analyzed, which excluded ATHOH8, AURKA, BUD31, CTSB, DSE, ESYT2, LGR4, RAN and RBBP4.

### Single guide RNA design and synthesis

sgRNAs directing the dCas9 effector domain fusion protein to the specific genomic site were designed to direct the methylome-modifying enzymes to positions around 33 base pairs upstream from their corresponding target CpG, because the dCas9-ED has been described to show highest epigenome modifying effectivity at 27 bp (±17 bp) from the PAM sequence of the sgRNA (Vojta et al., 2016). We designed two opposing sgRNAs per regulatory CpG, one binding to the sense and one binding to the anti-sense strand of the DNA. Furthermore, sgRNA target sites had a minimum of two mismatches to the next off-target site, to reduce off-targeting effects.

To evaluate gene knockdown through binding of the dCas9 without added effector domain to the transcription start site (TSS), we used the FANTOM5/CAGE online atlas (http://fantom.gsc.riken.jp/5/) to define the TSS of our target genes and selected a single sgRNA binding site at an average of 50 bp upstream of the TSS for optimal gene repression (Gilbert et al., 2014; Radzisheuskaya et al., 2016).

sgRNA expression plasmids were designed and synthesized following a previously published SAM target sgRNA cloning protocol (Konermann et al., 2015). In short, the sgRNA(MS2) cloning backbone (AddGene #61424) was digested with BbsI. Oligos representing the sgRNA target site with 20 bases in sense (Os) with a CACCG overhang and anti-sense (Oas) with an AAAC overhang were ordered from Eurofins and annealed. For genome reference, we used the UCSC Genome Browser on Human Feb. 2009 (GRCh37/hg19). Backbone and sgRNA defining insert were joined by a Golden Gate reaction. The resulting plasmid was expanded by bacterial transformation and assessed by sequencing.

### Stable dCas9-ED cell lines sgRNA transfection

HEK293 cells were transfected with the different sgRNA constructs by solid-phase reverse transfection as described (Neumann et al., 2010) but using Lipofectamine 2000 (Invitrogen #11668027) instead of Lipofectamine RNAiMAX.

### Immunostaining of HEK293 cells for DNA, sgRNA and dCas9-ED

HEK293 cells were fixed and stained at different time points between 3 and 9 days after transfection for the two components of the functional dCas9-ED by immunofluorescence staining. Cells were fixed with 4% PFA (Sigma-Aldrich #F8775) for 10 min in PBS with 0.5% Triton X-100 and blocked subsequently with 1% goat serum in PBS applied overnight. Mouse anti-Flag M2 monoclonal primary antibody (Sigma #F1804) and goat anti-mouse Alexa Fluor 568 secondary antibody (Invitrogen #A11004) were used to label the dCas9-ED. Successful transfection with the sgRNA plasmid was detected with rabbit anti-GFP monoclonal primary antibody (Cell Signaling Technology #2956, Danvers, MA) and goat anti-rabbit Alexa Fluor 488 (Molecular Probes #A11034, ). DNA was stained with DAPI.

### Confocal imaging of immunofluorescence stained epigenome targeted HEK293 cells

Confocal imaging was conducted using the Zeiss LSM 780 (Zeiss, Oberkochen, Germany) with the AutofocusScreen macro (24.02.2016; now see https://www-ellenberg.embl.de/resources/microscopyautomation), acquiring 25 Z-stacks per well with each comprising five slices per dCas9-ED-sgRNA combination (one dCas9-ED/one target gene). Each stack was acquired with a bright field image additionally to the DAPI (405 nm), sgRNA (488 nm) and dCas9-ED (568 nm) channels.

### 2D pre-screen of dCas9-ED HEK293 cells and phenotype evaluation

In a 2D pre-screen designed to select for significant methylome regulated target genes, a total of 129 possible dCas9-ED-sgRNA combinations were evaluated with an average of 6856 cells analyzed per combination. Solid-phase reverse transfection was used to deliver the sgRNA-expressing plasmid into the dCas9-ED expressing cell lines, where its expression was confirmed by GFP expression. We evaluated and classified the nuclear phenotype of cells expressing dCas9-ED and the sgRNA at 3, 5 and 7 days post transfection.

Raw HEK293 images of each sgRNA-dCas9-ED combination were smoothened by Gaussian convolution and single nuclei were segmented by Otsu thresholding. Single segments were further processed and split if necessary by segment erosion. Using the same CNN architecture as above, this time trained on annotated images of 2D HEK293 cells, single nuclei were classified by into cell cycle stages (inter-, pro-, meta-, anaphase) as well as significant phenotypes (macronuclei and apoptotic condensed DNA). Additionally, the transfection state of the cell was evaluated by the presence of dCas9-ED (M2-Flag) and sgRNA (GFP), and only cells expressing both components were included in the analysis.

Detected classes were further evaluated in comparison to non-targeted sgRNA transfected dCas9-ED cell lines as well as to non-transfected cells. All acquired time points were combined during analysis. Cells with a significantly higher (>1.5-fold) occurrence of a class compared to control cells were highlighted.

We found that only 18% of sgRNA-dCas9 combinations showed a significant effect on the mitotic phenotype, although 85% of those phenotypes correlated with the siRNA-induced phenotype and 88% correlated with knockdown phenotypes published in the online databases MitoCheck (Cai et al., 2018) and Cyclebase (Santos et al., 2015) (Fig. S4). The targeted CpG properties matched the expected gene regulatory effect of the dCas9-ED in only 8 out of 18 cases, suggesting that the majority of abnormal mitotic phenotypes were evoked by the dCas9 protein blocking access to regulatory sites.

## Supplementary Material

Supplementary information

Reviewer comments
