# Supplementary material for: Automated 3D light-sheet screening with high spatiotemporal resolution reveals mitotic phenotypes
Source: J Cell Sci. 2020 Jun 1;133(11):jcs245043. doi: 10.1242/jcs.245043 (PMC7286290; doi:10.1242/jcs.245043)
Supplement: Supplementary information [file joces-133-245043-s1.pdf]

1. Supplementary Figures

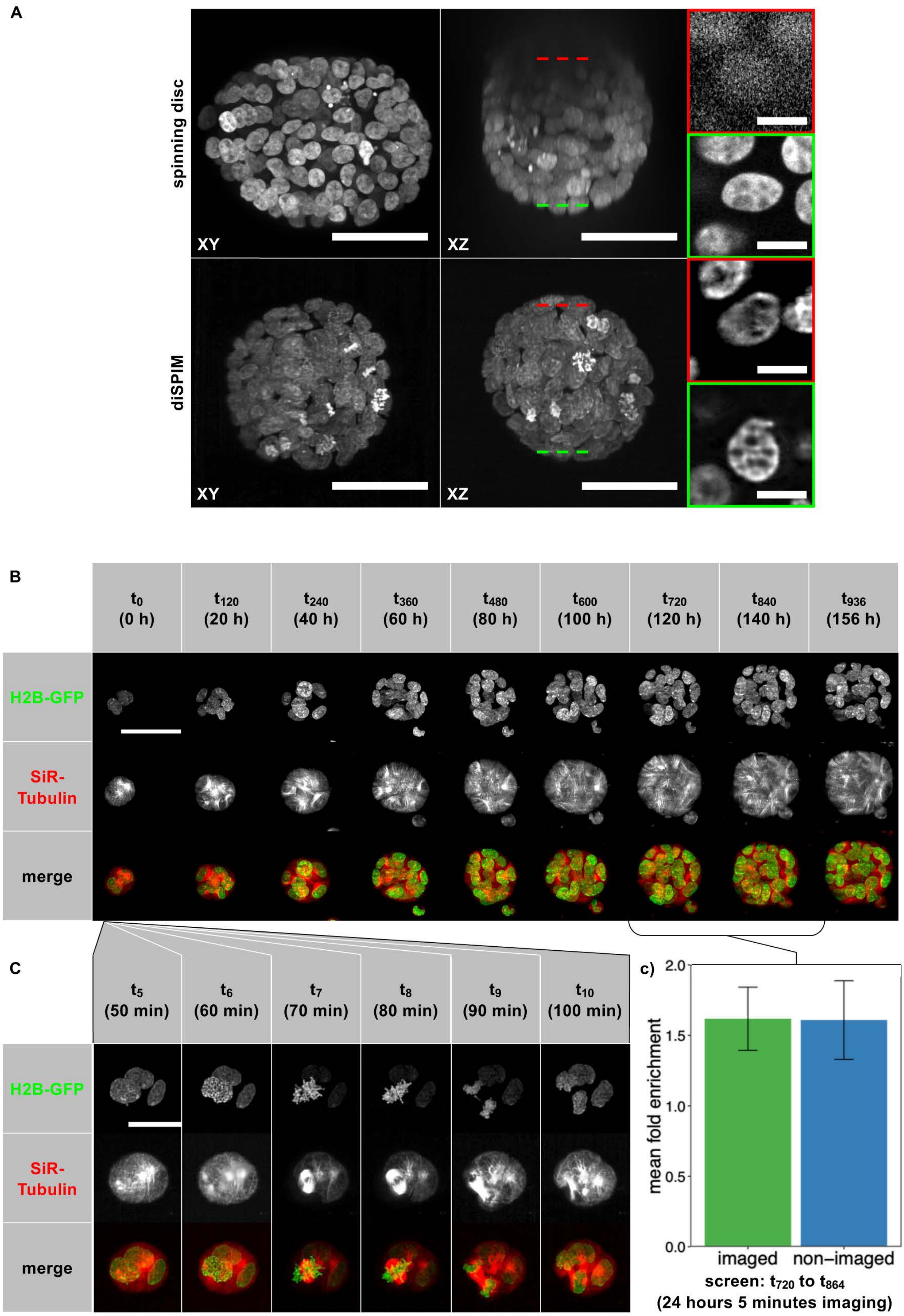

Fig. S1: High resolution and long term imaging capabilities of the diSPIM. (A) Direct comparison of spinning disc and light-sheet imaging performance. MCF10A H2B-GFP spheroids with a size of about

Journal of Cell Science • Supplementary information

80 µm in diameter were imaged six days after seeding single cells in Matrigel. XY and XZ maximum projections of the full 3D stack (scale bar = 50 µm) illustrate the XYZ resolution of the spinning disc and diSPIM microscopes. Inserts show single nuclei close to the detection objective (green box) or imaged 80 µm inside the sample (red box) in XY (scale bar = 10 µm), with the position of the corresponding Z-stack slices in the whole spheroid depicted by the red and green lines. (B) Example of an untreated MCF10A H2B-GFP spheroid imaged over 156 hours / 936 time points (t) every 10 minutes from the two-cell stadium to the fully developed spheroid in two channels (H2B-GFP and SiR-Tubulin dye). Scale bar = 50 µm. (C) High temporal and spatial resolution enable the detection of distinct features of the cytoskeleton and the different cell cycle stages (scale bar = 25 µm). (D) Mean fold enrichment of the number of nuclei during a 24 hour acquisition cycle for imaged and non-imaged spheroids (n_{imaged} = 31 / $n_{non\text{-}imaged}$ = 33). Error bars represent standard deviation.

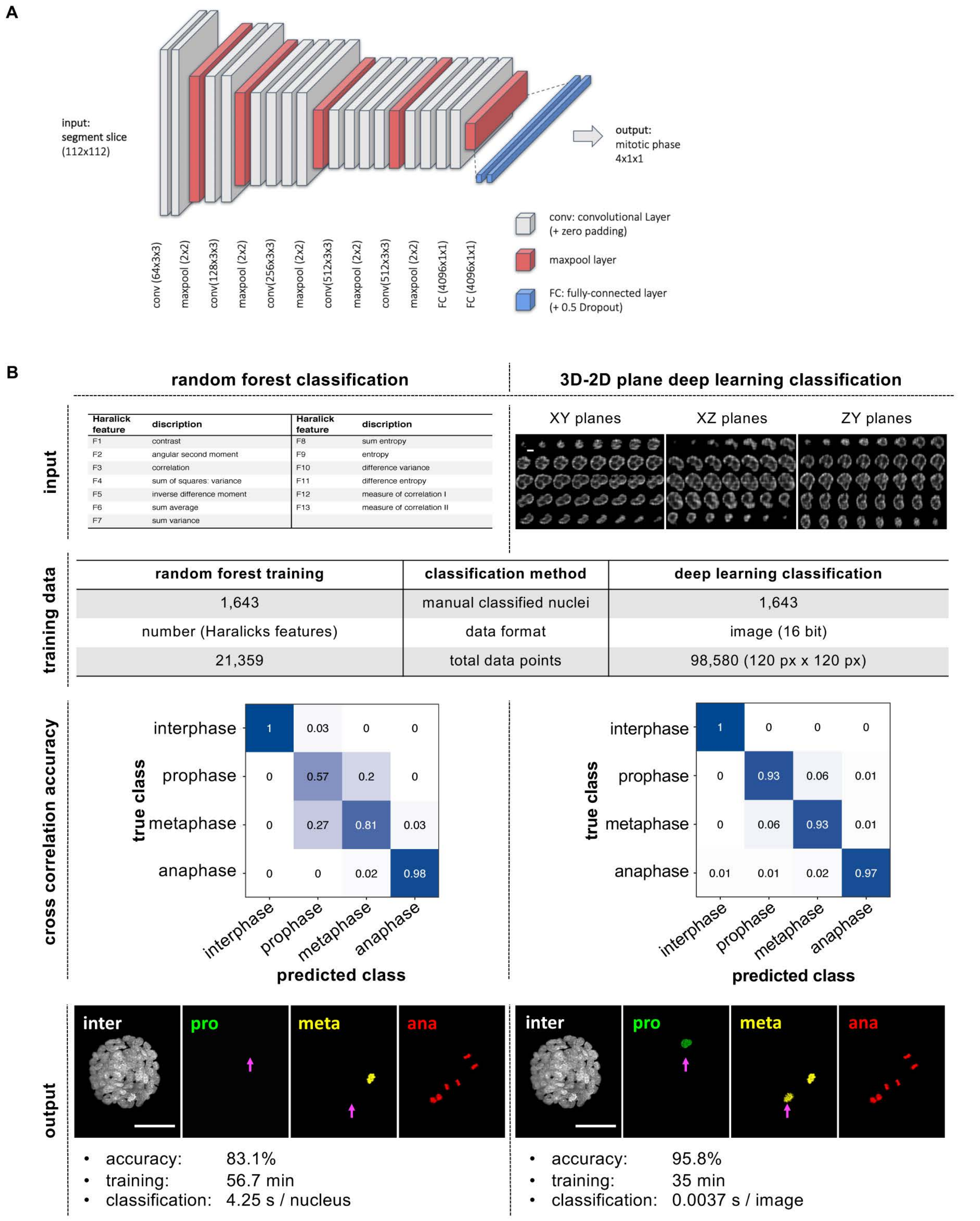

Fig. S2: Comparison of mitotic cell phase detection by a Random Forest classifier versus a Convolutional Neuronal Network. (A) The VGG-based convolutional neuronal network uses 2D image slices of the 3D segments (112 px x 112 px) as input. The network consists of convolutional and maxpooling layers as indicated, with the output combined by two fully connected layers, and outputs a probability for each of the cell cycle phases. (B) Comparison of the CNN with a Random Forest classifier. From top to bottom: Inputs were the 13 Haralick's features (F1-13) calculated by the 'hSPIM' data

Journal of Cell Science • Supplementary information

processing pipeline for random forest classification, and 2D (XY, XZ and YZ) slices of a 3D nucleus image for deep learning classification (scale bar = 5 μm). Manual labelling of the same nuclei resulted in a training data set comprising 21,359 data points for the RF classifier and 98,580 16-bit images for the CNN. Cross correlations are shown as measurements of classification accuracy. In direct comparison, classification differences between RF and CNN classification applied to the same image can be visually identified (magenta arrows; scale bar = 50 μm).

Journal of Cell Science • Supplementary information

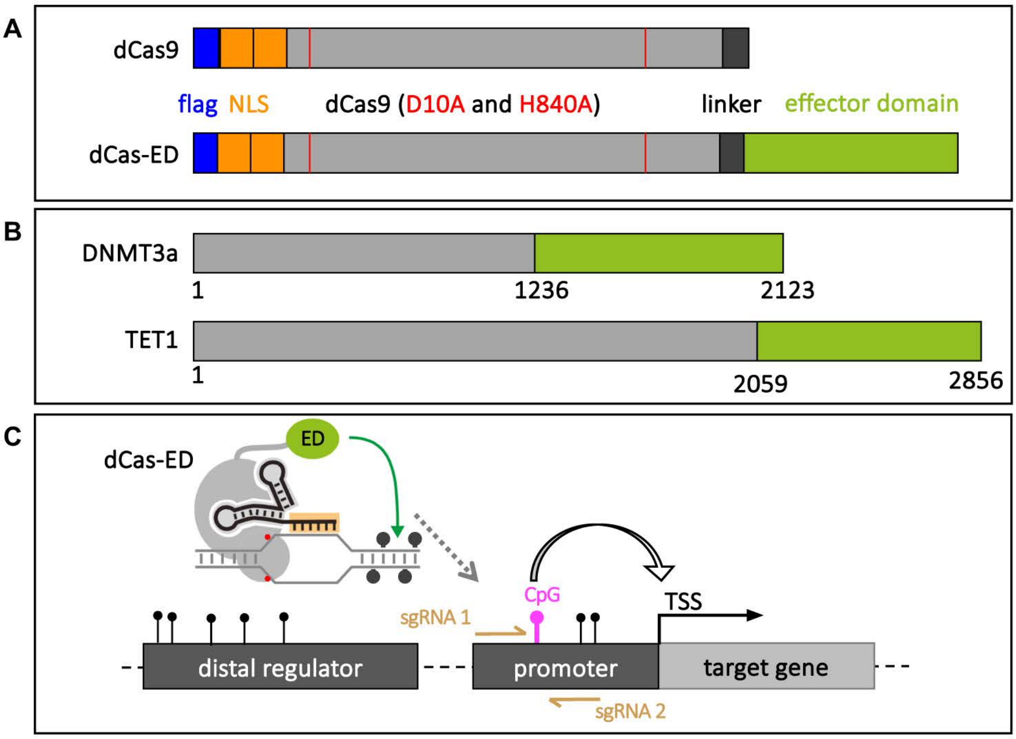

Fig. S3: dCas9-ED targeting regulatory CpGs. (A) CRISPR-Cas9 based epigenetic modifiers and binding control were composed of an M2-flag (blue), two nuclear localization sequences (orange), the dCas9 mutated at residue 10 and 840 (red) deactivating the endonuclease function of the Cas9, and the effector protein (green). The effector domains were fused C-terminally via a linker (dark grey). (B) Effector domains are the catalytically active, C-terminal domains of DNMT3A and TET1 from residue 1236 (DNMT3a) and 2059 (TET1) to the C-terminus of the protein. (C) CRISPR-dCas9 fused with the effector domain was located to specific target sites defined by the sgRNA. Combinations of dCas9-ED with sgRNA targeting correlated or anti-correlated CpGs defined gene regulatory properties. Per CpG, two sgRNAs with opposite orientation were transfected, targeting loci upstream and downstream of the CpG (magenta). CpGs were located in promotors or distal regulatory regions.

Journal of Cell Science • Supplementary information

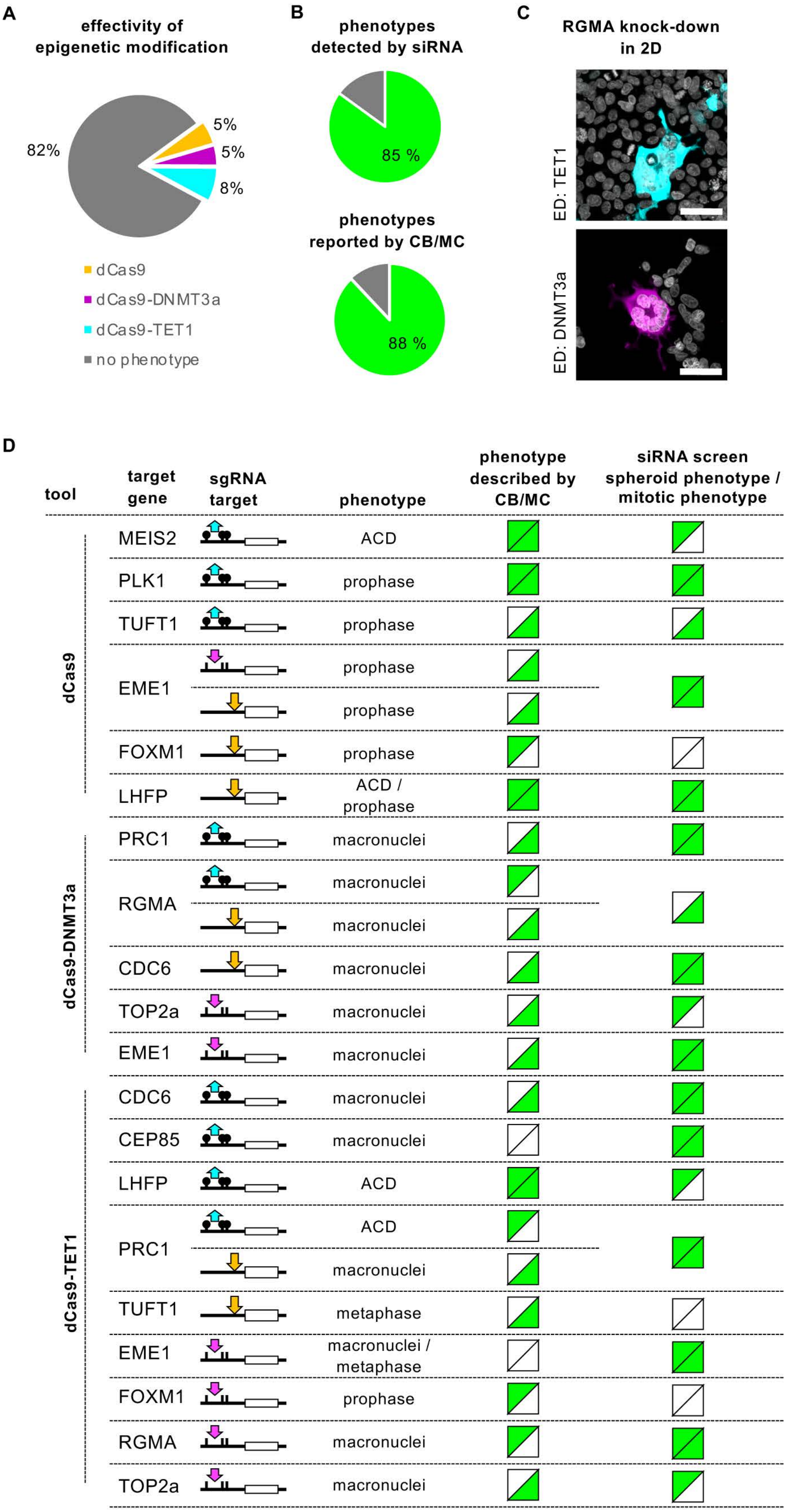

Fig. S4: Comparative analysis of knock-down mitotic phenotypes. (A) Fraction of dCas9, dCas9-DMT3a and dCas9-TET1 combinations with sgRNAs targeting expression of the 18 genes shown in d

Journal of Cell Science • Supplementary information

that resulted in a mitotic phenotype in HEK293 cells cultured in 2D. (B) Fraction of those phenotypes that was also detected in the siRNA screen (top) or reported in the Cyclebase (CB) and/or MitoCheck (MC) databases (bottom). (C) Example images of mitotic phenotypes evoked in 2D HEK293 cells upon dCas9-ED localization to anti-correlated (cyan) or correlated (magenta) regulatory CpGs. (D) List of target genes that showed a more than 1.5-fold increase in detection frequency of phenotypes (third column), such as increased representation of individual cell cycle phases or apoptotic condensed DNA (ACD), upon expression of dCas9 (top), dCas9-DNMT3a causing methylation (middle), or dCas9-TET1 causing demethylation (bottom) and transfection with sgRNA targeting anti-correlated (cyan) or correlated (magenta) regulatory CpGs or the transcription start site (orange) of the gene (second column). Detected phenotypes were compared with previously published databases (CB: Cyclebase, MC: MitoCheck) and siRNA diSPIM screen results. Green triangles indicate spheroid and mitotic phenotypes that were consistent across screens and sources.

Journal of Cell Science • Supplementary information

2. Supplementary Tables

Table S1: Selected target genes

Target genes were selected based on their association with the cell cycle, and correlation of their expression with the methylation level of either correlated or anti-correlated CpGs with high average absolute Pearson correlation value (R_{avg}).

Target gene	Name	Ambion siRNA #	Regulatory CpG	Relation of CpG methylation to gene expression	R_{avg}
**ATOH8**	Protein atonal homolog 8	s39645 / s39643	1	anti-correlated	0.52
**AURKA**	Aurora kinase A	s196 / s197	2	correlated / anti-correlated	0.52
**BUD31**	Protein BUD31 homolog	s17010 / s17009	1	correlated	0.45
**CDC6**	Cell division control protein 6	s2744 / s2746	2	anti-correlated	0.65
**CDCA5**	Sororin	s41424 / s41425	6	correlated	0.70
**CEP85**	Centrosomal protein of 85 kDa	s34959 / s34961	5	correlated / anti-correlated	0.64
**CTSB**	Cathepsin B	s3738 / s3739	1	anti-correlated	0.53
**DSE**	Dermatan Sulfate Epimerase	s26749 / s26750	1	anti-correlated	0.54
**EME1**	Essential Meiotic Structure-Specific Endonuclease 1	s44946 / s44945	1	correlated	0.66
**ESYT2**	Extended synaptotagmin-2	s33138 / s33136	2	anti-correlated	0.75
**F11R**	F11 Receptor	s27152 / s27151	6	anti-correlated	0.55
**FOXM1**	Forkhead Box M1	s5250 / s5249	1	correlated	0.68
**LGR4**	Leucine-Rich Repeat G Protein-Coupled Receptor 4	s30840 / s229314	1	anti-correlated	0.61
**LHFP**	Lipoma HMGIC Fusion Partner	s19847 / s19848	1	correlated	0.57
**LMNB2**	Lamin B2	s39477 / s39476	1	anti-correlated	0.60
**LRP1**	LDL Receptor Related Protein 1	s8278 / s8280	4	anti-correlated	0.70
**MAP7**	Ensconsin	s17263 / s17262	2	correlated	0.67

**MEIS2**	Meis Homeobox 2	s8666 / s8664	7	correlated / anti-correlated	0.57
**MYC**	Myc proto-oncogene protein	s9130 / s9131	2	anti-correlated	0.68
**PLK1**	Polo-like kinase 1	s448 / s450	4	correlated	0.64
**PRC1**	Protein regulator of cytokinesis 1	s17268 / s17269	1	anti-correlated	0.73
**RAN**	GTP-binding nuclear protein Ran	s11769 / s11768	1	anti-correlated	0.60
**RBBP4**	Histone-binding protein RBBP4	s55169 / s56872	1	anti-correlated	0.57
**RGMA**	Repulsive Guidance Molecule Family Member A	s32498 / s32500	7	correlated / anti-correlated	0.70
**TCF7**	Transcription factor 7	s13877 / s13878	2	anti-correlated	0.71
**TOP2A**	Topoisomerase II Alpha	s14307 / s14308	2	correlated	0.66
**TUFT1**	Tuftelin	s14510 / s14509	2	anti-correlated	0.59
**WBP1**	WW Domain Binding Protein 1	s24095 / s225969	1	correlated	0.52

Table S2: Comparison of spinning disc and diSPIM microscopy

Comparison of the acquisition properties and resulting image quality between a spinning disc microscope (Zeiss LSM 780) and the diSPIM system. Light-sheet imaging outperforms spinning disc microscopy in pixel resolution, acquisition speed, signal-to-noise ratio and phototoxicity.

	Spinning disc microscope	diSPIM
**XYZ stack (px x px x slices)**	1004 x 1002 x 233	2x (1024 x 1024 x 260)
**pixel resolution**	0.2 µm / px	0.1625 µm / px
**laser power**	1,320 µW	320 µW
**exposure / slice**	50 ms	1.75 ms
**stack acquisition duration**	53.2 s	4.5 s<br>(+ 15 s for image registration, merging and deconvolution)
**signal-to-noise ratio**	43.25	127
**avg. background signal**	18.4	0.941
**power density / phototoxicity**	168,000 W/cm^2	40,700 W/cm^2

Table S3: 23 features describing spheroid phenotypes

Feature	Name	Description	Global or nuclear feature
**1**	**spheroid growth rate (nuclei)**	rate of increase in nuclei count over the course of the time lapse	global
**2**	**prophase ratio**	fraction of nuclei classified as “prophase”	nuclear
**3**	**metaphase ratio**	fraction of nuclei classified as “metaphase”	nuclear
**4**	**anaphase ratio**	fraction of nuclei classified as “anaphase”	nuclear
**5**	**avg. cell volume**	average cell volume (in voxels) as ratio of spheroid volume to nuclei number	global
**6**	**prophase segment volume**	average nucleus size (in voxels) across all nuclei classified as “prophase”	nuclear
**7**	**metaphase segment volume**	average nucleus size (in voxels) across all nuclei classified as “metaphase”	nuclear
**8**	**anaphase segment volume**	average nucleus size (in voxels) across all nuclei classified as “anaphase”	nuclear
**9**	**interphase segment volume**	average nucleus size (in voxels) across all nuclei classified as “interphase”	nuclear
**10**	**spheroid volume**	spheroid volume (in voxels) throughout the time lapse	global
**11**	**avg. segment volume**	average nucleus size (in voxels) across all nuclei in all cell cycle phases	global
**12**	**spheroid growth rate (volume)**	rate of volume increase of the spheroid hull throughout the time lapse	global
**13**	**spheroid compactness**	factor describing the volume in relation to the largest extent	global
**14**	**convexity**	factor describing the volume in relation to the surface area	global
**15**	**nuclei migration speed**	average movement of all nuclei in 3D space in pixel per time point	global
**16**	**interphase transition duration**	average duration a nucleus spends in “interphase”	nuclear
**17**	**prophase transition duration**	average duration a nucleus spends in “prophase”	nuclear
**18**	**metaphase transition duration**	average duration a nucleus spends in “metaphase”	nuclear
**19**	**anaphase transition duration**	average duration a nucleus spends in “anaphase”	nuclear
**20**	**total number cell cycle transitions**	total number of deduced cell cycle phase transitions	global
**21**	**normal / abnormal transition**	fraction of cell cycle phase transitions that are biologically implausible	global
**22**	**spheroid roundness**	factor describing shape of spheroid	global
**23**	**size / spheroid roundness ratio**	ratio of spheroid volume to roundness	global

Table S4: Materials

Hardware

Workstation

hardware	supplier	description
CPU	Intel, Santa Clara, California, USA	i9-7980XE
GPU	NVIDIA, Santa Clara, California, USA	Titan xp 12 GB
Hard drive (RAID0)	Western Digital, San José, California, USA	WD-Red 8 TB
RAM	AMD, Santa Clara, California, USA	64 GByte DDR-4 PC2400
Motherboard	ASRock, Taipeh, Taiwan	X299 Taichi
Controller	Intel, Santa Clara, California, USA	SATA Controller, 10x 6 Gbit/s
Hard drive	Samsung, Seoul, South Korea	1 TB 960 Pro

ASI diSPIM hardware

hardware	supplier	description / number
camera cooling	Julabo	F250
quand filterset	AHF	F59-405<br>F73-410<br>F57-406
sCMOS cameras	Hamamatsu	ORCA-Flash4.0
laser	Spectral Applied Research	Laser Merge Module 5 (LMM5)

Software and workflows

Software

name	version	description
KNIME	3.5.5	Konstanz Information Miner
hSPIM	1.0	diSPIM raw image processing tool (‘hSPIM’)*
MicroManager	1.4	microscope control software
diSPIM plugin	NB_20180116	nightly build MicroManager diSPIM controller plugin

*hSPIM library available at *https://github.com/eilslabs/diSPIM_screen*

KNIME workflows (available at https://github.com/eilslabs/diSPIM_screen)

diSPIM_prescreen_stagescan_Pos_analysis
diSPIM_phenotype_screen_analysis_3D_spheroids
EpiTool_confocal_nuclei_classification
EpiTool_Class_quantitative_analysis

Haralick features used for phenotype characterization

Haralick feature	description	Haralick feature	description
F1	contrast	F8	sum entropy
F2	angular second moment	F9	entropy
F3	correlation	F10	difference variance

F4	sum of squares: variance	F11	difference entropy
F5	inverse difference moment	F12	measure of correlation I
F6	sum average	F13	measure of correlation II
F7	sum variance		

Source constructs

construct	source	description / number
#46911	AddGene	Gilbert_pHR-SFFV-dCas (Gilbert et al., 2014)
#71666	AddGene	pdCas9-DNMT3A-EGFP voj
#49792	AddGene	FH-TET1-pEF (*34*)
#61424	AddGene	sgRNA(MS2) cloning backbone

Antibodies

description	source	number	description
anti-Flag® M2	Sigma	F1804	primary mouse anti Flag M2 monoclonal antibody
anti-GFP	Cell Signaling Technology	2956	primary rabbit anti GFP monoclonal antibody
Anti-rabbit Alexa 488	Molecular Probes, Eugene, Oregon, USA	A11034	fluorescent secondary goat anti rabbit antibody
Anti-mouse Alexa 568	Invitrogen	A11004	fluorescent secondary goat anti mouse antibody

Primary and secondary antibodies were used at a dilution of 1:2000. HEK cells not expressing dCas9-ED were used as a negative control for the anti-Flag M2 antibody signal. The anti-GFP antibody was solely used to enhance the GFP signal after fixation for imaging.

Consumables and solutions

description	supplier	product number
beads: PS-Speck™ Microscope	ThermoFisher Scientific	P7220
Cell Culture Plate, 96-Well	Eppendorf, Hamburg, Germany	0030730119
CELLSTAR® OneWell Plate™	Greiner bio-one	670180
Cholera toxin	Sigma-Aldrich (Merck)	
Collagen type IV solution	Merck	C5533
culture flasks (25cm^2)	Greiner bio-one	
DAPI	Sigma-Aldrich (Merck)	D9542
DMEM/F12	ThermoFisher Scientific	11039
G418 (Geneticin)	Sigma-Aldrich (Merck)	4727878001
Gibson Assembly Master Mix	NEB, Ipswich, Massachusetts, USA	E2611
Insulin	Life Technologies	
Lipofectamine 2000	Invitrogen	11668027
Lipofectamine® RNAiMAX	ThermoFisher Scientific	13778075
Matrigel	Corning	354248
OptiMEM	ThermoFisher Scientific	51985026
PCR plate, 96 well	Kisker Biotech, Steinfurt, Germany	G060
trehalose dihydrate	Merck	T9531
trypsin	Life Technologies	25200056

Journal of Cell Science • Supplementary information
