# Supplementary material for: Automated 3D light-sheet screening with high spatiotemporal resolution reveals mitotic phenotypes
Source: J Cell Sci. 2020 Jun 1;133(11):jcs245043. doi: 10.1242/jcs.245043 (PMC7286290; doi:10.1242/jcs.245043)
Supplement: Reviewer comments [file joces-133-245043_review_history.pdf]

Journal of Cell Science

Automated 3D light-sheet screening with high spatiotemporal resolution reveals mitotic phenotypes

Bjöern Eismann, Teresa G. Krieger, Jürgen Beneke, Ruben Bulkescher, Lukas Adam, Holger Erfle, Carl Herrmann, Roland Eils and Christian Conrad
DOI: 10.1242/jcs.245043

Editor: Jennifer Lippincott-Schwartz

Review timeline

Original submission:	7 February 2020
Editorial decision:	2 March 2020
First revision received:	25 March 2020
Accepted:	30 March 2020

Original submission

First decision letter

MS ID#: JOCES/2020/245043

MS TITLE: Automated screening by 3D light-sheet microscopy with high spatial and temporal resolution reveals mitotic phenotypes

AUTHORS: Bjoern Eismann, Teresa Krieger, Juergen Beneke, Ruben Bulkescher, Lukas Adam, Holger Erfle, Carl Herrmann, Roland Eils, and Christian Conrad
ARTICLE TYPE: Tools and Resources

We have now reached a decision on the above manuscript.

To see the reviewer's reports and a copy of this decision letter, please go to: https://submit-jcs.biologists.org and click on the 'Manuscripts with Decisions' queue in the Author Area. (Corresponding author only has access to reviews.)

As you will see, the reviewer gave a favourable report but raised some critical points that will require amendments to your manuscript. I hope that you will be able to carry these out, because I would like to be able to accept your paper.

Please ensure that you clearly highlight all changes made in the revised manuscript. Please avoid using 'Tracked changes' in Word files as these are lost in PDF conversion.

I should be grateful if you would also provide a point-by-point response detailing how you have dealt with the points raised by the reviewers in the 'Response to Reviewers' box. Please attend to all of the reviewers' comments. If you do not agree with any of their criticisms or suggestions please explain clearly why this is so.

© 2020. Published by The Company of Biologists under the terms of the Creative Commons Attribution License (https://creativecommons.org/licenses/by/4.0/).

Reviewer 1

Advance summary and potential significance to field

The authors present a complete, convincing, and well-documented imaging method for screening mitotic phenotypes in a 3D cell culture model. This effort raises the bar for automated screening methods, taking effectively 2D + time methods into the 3rd dimension. Sophisticated machine learning assays are then used to quantitatively assess phenotypic changes.

Comments for the author

In this manuscript, Conrad and coauthors present an 'end-to-end' workflow for performing high-throughput screening of mitotic phenotypes in 3D cell cultures by light-sheet microscopy. Although I am not a biologist and so cannot comment on the biological findings, I am extremely impressed with what the authors have done on the technical front. The screening they perform is well-suited to the growing field of organoid biology and their paper very likely will become a classic for others to follow. I am especially happy with the quality of the data, the thorough explanations of methodological steps, the fact that their code is freely available, and the use of modern machine learning methods to conduct their image analysis. The comparison that is performed between CNNs and the more traditional random forest classification will also be useful to others in the field.

I have only minor suggestions for the authors:
Abstract
'dual inverted selective plane illumination microscope' - > 'dual-view inverted selective plane illumination microscope'
Introduction
'...standard 3D cell cultures at isotropic resolution.' It would be useful if the authors mention what spatial resolution they were actually able to achieve in practice. This could be measured using e.g. the microtubules in the SiR tubulin samples or estimated based on the use of Fourier ring correlation or related methods.
'diSPIM microscope' is redundant as the 'M' in 'diSPIM' stands for 'microscope'
I commend the authors for making their 'hSPIM' image processing code and KNIME workflow accessible. I would suggest highlighting this point in the main text of the manuscript.
In Fig. 1, the data images are missing scale bars.
What is the value of the scale bar in Fig. 2a?
The author's CNN takes projection slices (XY, XZ, ZY) as input. Did they consider inputting the entire deconvolved volume instead - I imagine this might yield even more accurate CNN results at the expense of computational slowdown/intractability. Please discuss this point.
Fig. 2g - y axis label for PLK1 seems to have an extra/dangling 'M'
I am a bit unclear how the 5 major phenotypic groups in Figure 3 were determined. Are these groupings qualitatively segregated?
Supplementary Table 2 - While I agree that the effective spatial resolution of diSPIM is likely higher than the spinning disk, the use of 'resolution' in this table is a bit misleading as the authors are using it to mean 'pixel resolution'. Please clarify this term in the table entry and caption. I am also unclear on the units for laser power - they are listed as uW / s. What is 's'?
Seconds? If so, this is an odd way to report power as as Watts are already energy per unit time. Presumably the power should not change over time...
Finally, what does the (+15 s) in Supplementary Table 2 refer to?
Low resolution pre-screen methods section,
'imaging plate' is spelled incorrectly as 'imagining plate'

© 2020. Published by The Company of Biologists under the terms of the Creative Commons Attribution License (https://creativecommons.org/licenses/by/4.0/).

First revision

Author response to reviewers' comments

Response to reviewer's comments:

Reviewer 1 Advance Summary and Potential Significance to Field:
The authors present a complete, convincing, and well-documented imaging method for screening mitotic phenotypes in a 3D cell culture model. This effort raises the bar for automated screening methods, taking effectively 2D + time methods into the 3rd dimension. Sophisticated machine learning assays are then used to quantitatively assess phenotypic changes.

We thank the reviewer for this favourable assessment of our manuscript.

Reviewer 1 Comments for the Author:
In this manuscript, Conrad and coauthors present an 'end-to-end' workflow for performing high-throughput screening of mitotic phenotypes in 3D cell cultures by light-sheet microscopy. Although I am not a biologist and so cannot comment on the biological findings, I am extremely impressed with what the authors have done on the technical front. The screening they perform is well-suited to the growing field of organoid biology and their paper very likely will become a classic for others to follow. I am especially happy with the quality of the data, the thorough explanations of methodological steps, the fact that their code is freely available, and the use of modern machine learning methods to conduct their image analysis. The comparison that is performed between CNNs and the more traditional random forest classification will also be useful to others in the field.

We greatly appreciate the reviewer's acknowledgement of the technical advances presented in our manuscript, and equally expect that they will be of use to colleagues in the field.

I have only minor suggestions for the authors:

Abstract
'dual inverted selective plane illumination microscope' - > 'dual-view inverted selective plane illumination microscope'

We have made this change in the Abstract and the main text, where the same wording appeared once.

Introduction
'...standard 3D cell cultures at isotropic resolution.' It would be useful if the authors mention what spatial resolution they were actually able to achieve in practice. This could be measured using e.g. the microtubules in the SiR tubulin samples or estimated based on the use of Fourier ring correlation or related methods.

The resolution (full width at half maximum or FWHM) was measured from the beads immersed in Matrigel acquired during the light-sheet screen of siRNA treated MCF10A spheroids. The point spread function (PSF) of 9 randomly selected beads from a fused and deconvolved hSPIM output image showed an average resolution of:
X = 937 nm
Y = 685 nm
Z = 877 nm

The measured FWHM values illustrate the advantage and disadvantage of using dual-view fusion. For view A alone, without fusion, the theoretical resolution based on the wavelength (488 nm) and the objective NA (0.8) would be 305 nm / 305 nm / 2028 nm in X / Y / Z. After dual-view image fusion, the measured Y resolution is a result of the high lateral resolutions contribution from view A and view B. Meanwhile, the lower X and Z resolutions result from the combination of a high lateral resolution and the low axial resolution from view A and view B, which after image fusion result in a

© 2020. Published by The Company of Biologists under the terms of the Creative Commons Attribution License (https://creativecommons.org/licenses/by/4.0/).

high axial resolution but reduced lateral resolution for the output image. The measured FWHM values show the improved axial but reduced lateral resolution resulting from dual view fusion.

'diSPIM microscope' is redundant as the 'M' in 'diSPIM' stands for 'microscope'

We thank the reviewer for pointing this out and have removed this redundancy throughout the manuscript.

I commend the authors for making their 'hSPIM' image processing code and KNIME workflow accessible. I would suggest highlighting this point in the main text of the manuscript.

As suggested, we have now added a comment in the Discussion section of the manuscript to highlight online availability: "Furthermore, we provide an easy-to-use image processing pipeline adapted to the geometry of the dual-view inverted light-sheet system, using a CNN for reliable cell cycle phase classification in 3D, which we have made available to the community online."

In Fig. 1, the data images are missing scale bars.

We thank the reviewer for pointing this out and have added the missing scale bars.

What is the value of the scale bar in Fig. 2a?

The value of this scale bar is 5 µm; we apologise for the omission and have added this information to the figure legend.

The author's CNN takes projection slices (XY, XZ, ZY) as input. Did they consider inputting the entire deconvolved volume instead - I imagine this might yield even more accurate CNN results at the expense of computational slowdown/intractability. Please discuss this point.

While classification of nuclei in 3D volumes would not have been impossible, creating an annotation of 3D segment masks for training would have been challenging based on the available data, so with 2D slices providing satisfactory results we proceeded with this implementation.

Fig. 2g - y axis label for PLK1 seems to have an extra/dangling 'M'

The extra 'M' was the result of an error during figure assembly; we thank the reviewer for spotting it and have removed it in the revised version.

I am a bit unclear how the 5 major phenotypic groups in Figure 3 were determined. Are these groupings qualitatively segregated?

Samples were segregated based on hierarchical clustering of all mitotic phenotypes, as described in the main text, figure legend and Methods section. The 5 phenotypic groups were then distinguished by cutting the hierarchical clustering tree at the corresponding height, with the number of groups chosen based on qualitative assessment of morphologic similarity of samples within groups. We have added this information to the figure legend and the Methods section.

Supplementary Table 2 - While I agree that the effective spatial resolution of diSPIM is likely higher than the spinning disk, the use of 'resolution' in this table is a bit misleading as the authors are using it to mean 'pixel resolution'. Please clarify this term in the table entry and caption.

We thank the reviewer for spotting this and have changed 'resolution' to 'pixel resolution' in the table entry and caption.

© 2020. Published by The Company of Biologists under the terms of the Creative Commons Attribution License (https://creativecommons.org/licenses/by/4.0/).

I am also unclear on the units for laser power - they are listed as uW / s. What is 's'? Seconds? If so, this is an odd way to report power as as Watts are already energy per unit time. Presumably the power should not change over time...

This was indeed an error in our manuscript; laser power was measured in uW , not uW / s. We apologise for the mistake and have corrected the unit of measurement to uW in the revised version.

Finally, what does the (+15 s) in Supplementary Table 2 refer to?

Acquisition of an image stack with the diSPIM took only 4.5 seconds; this was the time taken to produce the raw data. However, to compare results with the spinning disc microscope, a processing step (image registration, merging and deconvolution) was necessary, which required an additional 15 seconds of processing time. To clarify this, we have changed “+15 s” to “+ 15 s for image registration, merging and deconvolution” in the revised manuscript.

Low resolution pre-screen methods section, 'imaging plate' is spelled incorrectly as 'imagining plate'

We thank the reviewer for pointing out this error and have corrected it in the revised version of the manuscript.

Second decision letter

MS ID#: JOCES/2020/245043

MS TITLE: Automated screening by 3D light-sheet microscopy with high spatial and temporal resolution reveals mitotic phenotypes

AUTHORS: Bjoern Eismann, Teresa Krieger, Juergen Beneke, Ruben Bulkescher, Lukas Adam, Holger Erfle, Carl Herrmann, Roland Eils, and Christian Conrad
ARTICLE TYPE: Tools and Resources

I am happy to tell you that your manuscript has been accepted for publication in Journal of Cell Science, pending standard ethics checks.

© 2020. Published by The Company of Biologists under the terms of the Creative Commons Attribution License (https://creativecommons.org/licenses/by/4.0/).
